# Collagen turnover biomarkers and systemic right ventricle remodeling in adults with previous atrial switch procedure for transposition of the great arteries

**DOI:** 10.1371/journal.pone.0180629

**Published:** 2017-08-02

**Authors:** Magdalena Lipczyńska, Piotr Szymański, Magdalena Kumor, Anna Klisiewicz, Piotr Hoffman

**Affiliations:** 1 Adult Congenital Heart Disease Department, Institute of Cardiology, Warsaw, Poland; 2 Acquired Valve Disease Department, Institute of Cardiology, Warsaw, Poland; Bambino Gesù Children's Hospital, ITALY

## Abstract

**Background:**

Myocardial fibrosis is a potential pathophysiological mechanism leading to systemic right ventricular (SRV) deterioration. We hypothesize that circulating levels of collagen deposition markers are elevated in patients with SRV remodeling and this elevation may have a predictive value.

**Methods:**

We prospectively evaluated 56 patients with D-TGA after the atrial switch procedure (mean age 25.6 ± 4.8, range 18–37 years; 67% males). Serum levels of procollagen type III amino-terminal propeptide (PIIINP), collagen type I carboxy-terminal telopeptide (CITP), procollagen type I N-terminal propeptide (PINP), matrix metalloproteinase (MMP 1, MMP 9) and a tissue inhibitor of matrix metalloproteinase (TIMP 1) and N-terminal pro-brain natriuretic peptide (NT-pro-BNP) were measured and compared with healthy controls. The relationship between these serum markers, echocardiographic and cardiac magnetic resonance parameters and the outcome at a follow-up of 61 months (range, 24–85 months) was determined.

**Results:**

Compared with the healthy control group, the study group had significantly higher levels of TIMP1, PIIINP, CITP, PINP and NT-pro-BNP (p<0.05, each). The levels of PIIINP and CITP were significantly higher among patients with an SRV mass index above the mean value. The level of PIIINP was significantly higher among patients with an SRV EDV index above the mean value. CITP was significantly elevated in SRV late gadolinium enhanced (LGE) positive patients, compared to patients without SRV LGE. MMP9 and TIMP1 predicted an adverse clinical outcome on univariate Cox proportional hazard survival analysis in addition to well proven predictors of outcome (SRV EF and NYHA).

**Conclusions:**

We demonstrated a pattern of altered collagen turnover adversely related with the indices of SRV remodeling and an adverse clinical outcome in patients with SRV.

## Introduction

D-transposition of the great arteries (D-TGA) is one of the most common, severe cyanotic congenital heart diseases. [1] The arterial switch operation is currently the procedure of choice. [2] Before the introduction of anatomical correction, D-TGA was repaired with the atrial switch procedure by using the Mustard or Senning technique, which revolutionized the outcome, allowing patients to survive well into adulthood. [3, 4] Importantly, in this type of operation the right ventricle (RV) is in the systemic position and subjected to significant pressure overload. [5] The cumulative survival rate after the atrial switch is about 80% after 25 years. [6, 7, 8] Now, a considerable number of these surviving patients are adults and provide a challenge to clinicians. There are some specific long-term complications associated with increased mortality and morbidity. [9, 10] Systemic right ventricular (SRV) dysfunction has been identified as a leading problem in the long term, suggesting the limited capacity of the morphologically RV while coping with systemic circulation. [11]

Myocardial fibrosis is a potential pathophysiological mechanism leading to a systemic ventricular deterioration. The late gadolinium enhancement cardiovascular magnetic resonance (LGE CMR) imaging of the SRV is able to visualize focal fibrosis. However, data on the incidence of LGE positive results among patients with SRV are ambiguous. [12–14] Some authors described the presence of diffuse fibrosis in various patients with congenital heart disease, also in those with systemic right ventricle, and correlated it with ventricular volume and function. [15] Measuring interstitial expansion in the free wall of the systemic right ventricle is difficult. [16] Myocardial intestinal collagen content, apart from being directly measured by endocardial biopsy, can now also be assessed with serum analysis of the breakdown products of collagen I and collagen III. According to previous reports, serological markers of collage type I and III turnover such as procollagen type III amino-terminal propeptide (PIIINP), collagen type I carboxy-terminal telopeptide (CITP) and procollagen type I N-terminal propeptide (PINP) might serve as surrogate estimates approximating the intensity of fibrosis occurring in the myocardium. [17] Enzymes that control collagen turnover matrix metalloproteinase (MMP) and a tissue inhibitor of matrix metalloproteinase (TIMP) can also be measured with serum analysis. [17–19] It may be hypothesized that, circulating levels of collagen deposition markers would be elevated in patients with SRV remodeling. Dos et al., in a small study, showed altered collagen turnover biomarker (CTB) levels in patients with SRV, but did not acknowledge a correlation between CTB and the function of the systemic ventricle. [20] Therefore, our study was designed to assess the relation between myocardial fibrosis, estimated indirectly by CTB, and SRV remodeling of patients with D-TGA after the atrial switch and its potential prognostic value.

## Materials and methods

### Study population

This was a prospective cross-sectional study. Fifty-six adult patients (mean age 25.6 ± 4.8, range 18–37 years; 67% males) with D-TGA after the atrial switch procedure, attending the adult congenital heart disease outpatient clinic at our institution, were screened between 1 June 2010 and 31 December 2011 for possible participation in the study. Patients with active inflammation, chronic inflammatory disease, conditions known to alter collagen turnover including chronic liver disease, connective tissue disease, metabolic bone disease, malignancy, severe chronic kidney disease (estimated glomerular filtration rate < 30 ml/Kg/min) or patients after recent trauma or surgery (< 6 months) were excluded from the study. The control group consisted of 29 healthy staff members at our institution. Comparisons between healthy volunteers and patients were limited to biomarkers assessment. All subjects gave written informed consent to participate in the study. The study protocol was approved by the local Institutional Review Board. Ethics Committee of the Institute of Cardiology approved this study.

### Blood assays

Peripheral venous blood samples were obtained during clinical assessment and centrifuged at 4°C at 2500 g. Samples were aliquoted and stored at– 70°C until analysis (less than 6 months). Serum concentration of C-terminal telopeptide of type I collagen (CITP) was assayed using the the UniQ CITP RIA kit. The sensitivity of the assay was about 0.4 g/L. Intra- and inter-assay CV was 6.3 and 9.0%, respectively. Serum concentration of PINP was determined with the UniQ PINP RIA kit. Sensitivity of the assay was 2.0 μg/L. Intra- and inter-assay CV were 10.2 and 9.8%, respectively. For the determination of PIIINP the UniQ PIIINP RIA kit was applied. Sensitivity of the assay was 0.3 μg/L. Intra- and inter-assay CV were 7.0 and 7.2%, respectively. The UniQ RIA kits were obtained from Orion Diagnostica Oy, Espoo, Finland. Concentration of the MMPs and TIMP-1 were assayed using the quantitative sandwich enzyme immunoassay technique. All kits were purchased from R&D Systems, Inc., Minneapolis, MN, USA. Sensitivity for MMP-2 was 0.047 ng/mL, intra- and inter-assay CV were 5.4 and 6.9% respectively. Sensitivity for MMP-9 was 0.156 ng/mL, intra- and inter-assay CV were 2.9 and 7.8% respectively. Sensitivity for TIMP-1 was 0.08 ng/mL, intra- and inter-assay CV were 5.0 and 4.9% respectively. N-terminal brain natriuretic peptide (NT-pro-BNP) was determined using an immunoradiometric assay (Cobas e 601 analyzer, Roche Diagnostics, Germany). Aldosterone concentration in the serum samples was quantified using the ALDOCTK-2 RIA (Radio ImmunoAssay) kit from DiaSorin Inc. Stillwater, MN, USA.

### Echocardiography

All patients underwent a complete transthoracic echocardiographic study (Vivid 9; GE Healthcare, Milwaukee, WI) by a cardiologist with experience in congenital heart disease (ML) blinded to the clinical data. A cine loop of at least three consecutive heartbeats was stored digitally for off-line analysis. Echopac software (GE Vingmed, Horten, Norway) was used to analyze the stored data. Right ventricular function was assessed in a modified apical four-chamber view, with a minimization of sector width and a slight tilt of the transducer if needed to optimize SRV visualization. Gray scale images were performed with frame rates *>*60 frames per second (fps). The following standard echocardiographic parameters were measured offline: (1) the SRV diastolic diameter just below the tricuspid valve, (2) the SRV fractional area change (FAC), (3) the tricuspid annular plane systolic excursion (TAPSE), (4) s’ velocities at the lateral tricuspid annulus using pulsed-wave tissue Doppler with >140 fps. [21] Conventional color Doppler imaging was used to quantify the severity of tricuspid valve regurgitation. The severity of tricuspid regurgitation was defined based on comprehensive Doppler assessment. [22] Right ventricular global longitudinal deformation was measured by speckle-tracking analysis in the apical four-chamber view. To determine the SRV peak systolic global longitudinal strain, the endocardial border of the right ventricle was traced manually and tracked with the appropriate software. The utility and particular methodology of measurements were published elsewhere. [23, 24]

### Cardiac magnetic resonance

CMR studies were performed with a 1.5T scanner (Avanto, Siemens, Erlangen, Germany). The CMR protocol included cine steady-state free precession (SSFP) breath hold sequences in 2-, 3-, and 4-chamber views and a stack of short axis images (slice of thickness 8–10 mm) acquired parallel to the atrioventricular groove from base to apex. The SRV endocardial and epicardial borders were outlined manually at end-diastole and end-systole. On the basis of these data, analysis of SRV volumes and the ejection fraction were obtained by using dedicated software (Mass 6.2.1, Medis, Leiden, The Netherlands). Approximately 10–15 min after the administration of 0.2 mmol/kg body weight gadolinium-diethylenetriaminepentaacetate (Magnevist, Schering, Germany), a late enhancement study using a 3D spoiled turbo Gradient Echo sequence with a selective 180° inversion recovery pre-pulse was acquired in the short and long axis of the left ventricle. Inversion times were individually selected using a Look-Locker sequence to optimally null the signal of normal myocardium. Post-acquisition analysis of ventricular size and function as well as LGE were done using commercially available software packages. Right ventricular fibrosis was assessed in a binary way—as either present or absent.

We routinely used CMR for the assessment of the systemic right ventricle, therefore only patients not eligible for CMR due to: (1) implanted pacemakers, (2) a mental handicap precluding cooperation during CMR, (3) pregnancy and those who (4) did not consent to undergo CMR, were excluded from the study. Based on these criteria 15 out of 56 patients did not take part in the CMR study, including 3 patients who declined to participate on personal grounds (mainly due to concerns about gadolinium toxicity and the potential inconvenience associated with the study), and 8 who were excluded because of an implanted pacemaker and 4 because of a mental disorder.

### Follow up

The cardiologist working in the adult congenital heart disease outpatient clinic at our institution prospectively followed up all patients. A combined adverse clinical end point was defined as: (1) an increase in the NYHA class to ≥ 3, (2) the occurrence of symptomatic arrhythmia requiring medical or interventional therapy, (3) death. Data on clinical events was collected during (1) regular or unplanned (attributable to new signs or symptoms) visits at our institution, and (2) hospitalizations.

### Statistical analysis

Continuous data are presented as the mean ± SD. Categorical data are presented as counts and percentages. The normal distribution of data was assessed using the Kolmogorov-Smirnov test. Depending on the distribution, comparisons between groups were made by the Student t test for unpaired data for continuous variables and by the chi-square test for categorical variables or by the Mann-Whitney test, for not normally distributed variables. Pearson or Spearman correlation coefficients were used according to the distribution type identified for each variable. The univariate logistic regression analysis was performed to test the predictive value of selected parameters. The ability of clinical, CMR and echocardiographic parameters to predict adverse clinical outcomes, was assessed by univariate Cox proportional—hazard survival analysis. The concentrations of NT-pro-BNP and aldosterone were transformed with natural logarithm, as they were not normally distributed. The statistical significance was set at p *<* 0.05.

Analyses were performed using the SPSS version 16 (StataCorp LP, College Station, TX).

## Results

Fifty-six patients with D-TGA following the atrial switch procedure and 29 healthy age and sex matched controls were prospectively evaluated. The basic clinical characteristics as well as the results of the CMR and the echocardiographic evaluation of the SRV in D-TGA patients are presented in Table 1.

**Table 1:** Baseline characteristics of the studied group.

	D-TGA (n = 56)
**Age, yrs**	25.6 ± 4.8
**Male, n (%)**	37 (67)
**Race, white n (%)**	56 (100)
**Age at operation, mths**	29.2 ± 2.5
**Senning/Mustard, n (%)**	46 (82)/10 (18)
**NYHA, n (%)**	
I	44 (79)
II	11 (20)
III	1 (0.5)
**Echocardiography**	
TAPSE, mm	13.3 ± 2.7
SRV s’, cm/s	9.1 ± 2.2
SRV FAC, %	31.5 ± 7.7
SRV GLS, %	15.4 ± 2.8
CMR*	
SRV EF, %	48.0 ± 9.7
SRV mass index, g/m²	38.5 ± 14.7
SRVEDV index, ml/m²	125.2 ± 32.8
SRV LGE positive, n %	4 (10)

*CMR was performed on 41 patients.

NYHA—New York Heart Association, TAPSE—tricuspid annular plane systolic excursion, SRV—systemic right ventricle, FAC—fractional area change, GLS—global longitudinal strain, CMR—cardiac magnetic resonance, EF—ejection fraction, EDV—end diastolic volume, LGE—late gadolinium enhancement.

### Comparisons with healthy controls

There was no difference between D-TGA patients and controls in terms of age and sex (mean age 25.6 ± 4.8 years vs 27.1 ± 5.9 years, p = 0.1 and 67% male vs 66% male, p = 0.9, respectively) Compared with the healthy control group, the study group had significantly higher levels of TIMP1, PIIINP, CITP, PINP and NT-pro-BNP (Table 2). There was no significant difference in MMP2, MMP9 and aldosterone levels. We did not find any correlations between the levels of the different CTBs.

**Table 2:** Comparison of biochemical markers between the studied group and healthy controls.

	D-TGA patients	Healthy controls	P Value
**MMP2, ng/ml**	202.9 ± 37.2	214.7 ± 14.1	0.1
**MMP9, ng/ml**	463.3 ± 263.3	403.9 ± 244.5	0.3
**TIMP1, ng/ml**	153.3 ± 36.5	135.1 ± 34.2	0.03
**PIIINP, μg/l**	4.8 ± 1.5	3.3 ± 0.5	< 0.0001
**CITP, μg/l**	4.0 ± 1.4	3.1 ± 1.1	0.007
**PINP, μg/l**	62.5 ± 28.5	50.3 ± 12.9	0.03
NT-pro-BNP, pg/ml**	176.5 (IQR 198.3)	42.7 (IQR 56.6)	0.0001*
Aldosterone (pmol/l)**	141.5 (IQR 89.5)	198.5 (IQR 211.65)	0.1*

MMP- matrix metalloproteinase, TIMP- tissue inhibitor of matrix metalloproteinase, PIIINP—procollagen type III amino-terminal propeptide, CITP—collagen type I carboxy-terminal telopeptide, PINP—procollagen type I N-terminal propeptide, NT-pro-BNP—N-terminal pro-brain natriuretic peptide.

* P value for Mann-Whitney nonparametric test.

** Median (Interquartile Range, IQR) for non-normally distributed data.

We found no relation between sex and the concentrations of serum fibrosis markers either in patients or in controls. There was a correlation between CTB levels and age in D-TGA group for: PIIINP (r = − 0.53, p<0.001), CITP (r = − 0.44, p = 0.001), and PINP (r = − 0.52, p<0.001), but not in healthy controls.

### Biomarker levels and SRV function—Echocardiography

**Fig 1:**
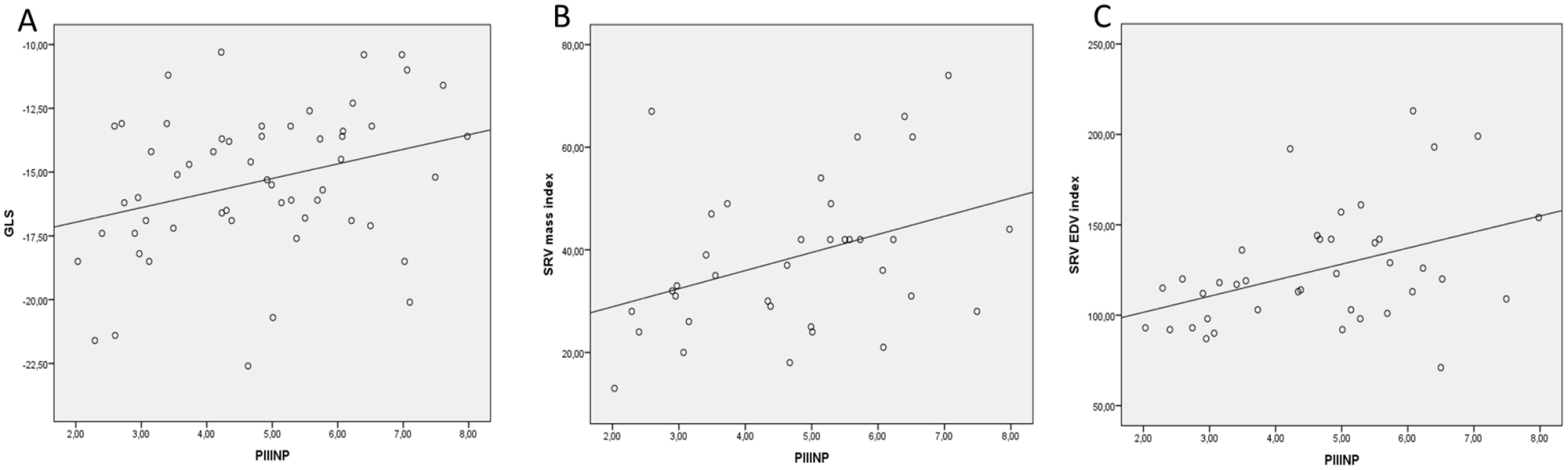
Relationship between PIIINP levels and SVR function and remodeling parameters. (A) Correlation between PIIINP and SRV GLS (r = 0.31, p = 0.01), (B) correlation between PIIINP and SRV mass index (r = 0.38, p = 0.02), (C) correlation between PIIINP and SRV EDV index (r = 0.42, p = 0.008).

The levels of PIIINP and NT-pro-BNP were significantly higher among patients with low GLS (> − 13%) (5.9 ± 1.4 μg/l vs 4.5 ± 1.5 μg/l, p = 0.02, 861.2 ± 956.3 pg/ml vs 225.3 ± 183.9 pg/ml, p<0.001, respectively) There was a weak correlation between SRV GLS and TIMP1 (r = 0.27, p = 0.04), PIIINP (r = 0.31, p = 0.01) and CITP (r = 0.31, p = 0.01) and no correlation between standard echocardiographic parameters of systolic SRV function (SRV s’, TAPSE, SRV FAC) and any of the CTB levels (Fig 1A). On univariate logistic regression analysis PINP (odds ratio 1.03, 95% confidence interval 1.0–1.05; p = 0.04), PIIINP (1.87, 1.05–3.33; p = 0.03) and NT-pro-BNP (1.004, 1.001–1.007, p = 0.02) were predictors of low GLS defined as GLS > − 13%.

### Biomarker levels and CMR SRV parameters

The levels of PIIINP and CITP were significantly higher among patients with an RV mass index above the mean value (5.3 ± 1.4 μg/l vs 4.1 ± 1.5 μg/l, p = 0.03, 4.7 ± 1.4 μg/l vs 3.6 ± 1.4 μg/l, p = 0.03 respectively). There was a positive weak-to-moderate correlation between the RV mass index and PIIINP (r = 0.38, p = 0.02), CITP (r = 0.43, p = 0.009) and PINP (r = 0.38, p = 0.02) (Fig 1B). Neither aldosterone nor NT-pro-BNP correlated with the RV mass index. On univariate logistic regression analysis PIIINP (0.59, 0.36–0.96, p = 0.003) and CITP (0.57, 0.33–0.98, p = 0.04) were predictors of an RV mass index above the mean value.

The level of PIIINP was significantly higher among patients with an RV EDV index above the mean value (5.5 ± 1.1 μg/l vs 4.1 ± 1.5 μg/l, p = 0.005). Right ventricular end-diastolic volume index correlated positively only with PIIINP (r = 0.42, p = 0.008) (Fig 1C).

On univariate logistic regression analysis PIIINP (0.5, 0.29–0.85, p = 0.01) was the only predictor of an enlarged SRV cavity (SRV EDV index > 125 ml/m^2^).

Neither CTB levels nor the aldosterone level differed significantly in patients with preserved vs impaired SRV systolic function defined as CMR SRV EF < 45%. There was a significant difference in NT-pro-BNP levels, 162,6 pg/ml vs 308,0 pg/ml respectively (p = 0.01). There was no correlation between NT-pro-BNP and SRVEF.

On univariate logistic regression analysis TIMP 1 (0.96, 0.93–0.99; p = 0.02) was the only predictor of reduced SRV systolic function (CMR SRVEF < 45%).

### Biomarker levels and LGE

In 4 (10%) out of 41 (73%) patients in whom CMR studies were obtained, the small foci of LGE were detected. In all cases they were located at the insertion between the SRV and the interventricular septum. These patients had significantly lower SRV EF (45% vs 48%, p = 0.01), a larger SRV cavity (end diastolic volume index 144.1 ml/m^2^ vs 123.4 ml/m^2^, p = 0.01) and a higher SRV mass index (47.2 g/m^2^ vs 37.3 g/m^2^, p = 0.01), compared to patients without LGE. CITP was significantly elevated in SRV LGE positive patients, compared to patients without RV LGE (5.0 ± 2.7 μg/L vs 3.9 ± 1.3 μg/L, p = 0.008). Other biochemical markers of collagen turnover did not achieve significant difference. NT-pro-BNP was increased in LGE positive patients but didn’t reach statistical significance (629.4 [IQR 1559.8] pg/ml vs 169.9 [IQR 156.8] pg/ml, p = 0.08). Aldosterone levels were not elevated in SRV LGE positive compared to LGE negative patients. Neither CTB parameters nor NT-pro-BNP and aldosterone predicted positive LGE results on univariate regression analysis.

### Value of CTB in predicting adverse clinical events

During a median follow-up time of 61 months (range 24–85 months), 12 patients (21%) reached the specified composite end point. There were 2 sudden cardiac deaths, 4 patients progressively deteriorated to NYHA class 3 or 4, and 6 had clinically relevant arrhythmias (all supraventricular, requiring cardioversion). The median time between the study entry and the occurrence of the composite endpoint was 32 ± 21 months. On univariate Cox proportional hazard survival analysis MMP9, TIMP1, SRV EF and NYHA class ≥ 2 predicted an adverse clinical outcome (Table 3).

**Table 3:** Univariate predictors of adverse clinical events.

Variable	Hazard ratio (95% CI)	P value
**Biochemical parameters**		
(ln) NT-pro-BNP	1.001 (1.00–1.001)	0.23
(ln) aldosterone	1.00 (0.99–1.002)	0.83
MMP2	1.003 (0.99–1.01)	0.63
MMP9	1.004 (1.001–1.006)	0.01
TIMP1	1.02 (1.005–1.035)	0.008
PIIINP	1.12 (0.79–1.58)	0.52
CITP	1.28 (0.87–1.88)	0.19
PINP	0.99 (0.97–1.01)	0.86
**CMR parameters**		
SRV EF	0.90 (0.83–0.97)	0.007
SRV LGE positive	1.19 (0.24–5.83)	0.83
**Echocardiographic parameter**		
SRV GLS	1.14 (0.92–1.41)	0.21
**Clinical parameters**		
Age	0.98 (0.88–1.11)	0.85
Age at operation	0.93 (0.73–1.19)	0.57
NYHA class ≥ 2	0.13 (0.04–0.39)	0.001

MMP- matrix metalloproteinase, TIMP- tissue inhibitor of matrix metalloproteinase, PIIINP—procollagen type III amino-terminal propeptide, CITP—collagen type I carboxy-terminal telopeptide, PINP—procollagen type I N-terminal propeptide, NT-pro-BNP—N-terminal pro-brain natriuretic peptide, CMR—cardiac magnetic resonance, SRV—systemic right ventricle, EF—ejection fraction, LGE—late gadolinium enhancement, GLS—global longitudinal strain, NYHA—New York Heart Association, ln—natural logarithm.

## Discussion

This is the second paper concerning CTB among patients with D-TGA after the atrial switch operation. [20] In a prospective study with a larger group and a wider profile of collagen turnover markers, we demonstrated an increased fibrotic signal (PIIINP, PINP, CITP) as well as an increased matrix turnover signal (MMP9, TIMP1) in these patients, as compared to age- and gender-matched healthy controls. The data suggest that some of CTB may have a predictive value in this group of patients.

There is growing evidence that myocardial fibrosis may be a pathophysiological substrate behind SRV dysfunction. [25] Although tissue biopsy is the gold standard for the diagnosis of myocardial fibrosis, a number of circulating biomarkers have been proposed for the noninvasive assessment of this lesion. [26]

The only study concerning SRV done by Dos et al., even in a population showing a good baseline profile in terms of SRV mass (57.4 ± 17 g/m2), ejection fraction (54.9 ± 7.5%) and clinical condition (100% NYHA class I or II) exhibits a pattern of altered collagen turnover (CITP, CICP) and neurohormonal (NT-pro-BNP) activation. [20] Compared to the healthy controls, we also found significantly higher levels of TIMP1, PIIINP, CITP, PINP and NT-pro-BNP among patients with SRV.

Enhanced signals from tissue growth factors and angiotensin-II induce the production of PIIINP, which is the most frequently and extensively studied marker of tissue fibrogenesis. PIIINP is cleaved off during conversion from type III procollagen to type III collagen in the fibroblasts, and is subsequently released into the bloodstream. [27] There is growing data presenting elevated levels of PIIINP in patients with heart failure, hypertension or coronary heart disease but this literature is based on patients with a systemic left ventricle. Our data suggests that among CTB, mostly PIIINP may be a good indicator for SRV remodeling and for reduced longitudinal systolic function. It is noteworthy that although our patients exhibited a pattern of altered collagen turnover, they were in good clinical condition (99% in NYHA class I or II). Sugimoto et al. proved increased serum PIIINP levels in proportion to the severity of ventricular volume load among patients with ventricular or atrial septal defects. [28] We also found a positive correlation between the PIIINP level and the SRV cavity size, which is a surrogate of volume load. The right ventricle in the systemic position is coupling with systemic circulation, which also means that there is a pressure overload resulting in the SRV mass increasing. We prove the correlation between the PIIINP and SRV mass index. PIIINP and CITP were also predictors of a higher SRV mass. Similar results connecting RV pressure overload with CTB activation comes from two consecutive studies. Sugimoto et al. show the correlation between the pressure gradient across the pulmonary valve and the PIIINP level. [28] Circulating levels of PIIINP, CITP, MMP-9, and TIMP-1 were found to be higher in pulmonary arterial hypertension as compared with age- and sex-matched healthy controls and PIIINP correlated with the severity of disease in the study done by Safdar et al. [29]

We assessed associations of biomarker levels with CMR parameters of myocardial fibrosis. The results of our study indicating augmented fibrosis in SRV patients are consistent with several previously reported data demonstrating ventricular myocardial fibrosis indicated by LGE of MRI. [12, 30] Using CMR imaging, Babu-Narayan et al reported that LGE positive patients have poorer systemic right ventricular function and a worse prognosis. [12] Rydman et al. showed that SRV fibrosis in patients with transposition of the great arteries post atrial redirection surgery is common and is associated independently with a clinical outcome. [31] Broberg et al noted similar phenomenon in correlation with diffuse fibrosis measured by equilibrium contrast CMR in a heterogenous group of CHD patients. The fibrosis index was particularly high in patients with SRV and there was a positive relationship between end diastolic volume index and the fibrosis index and a trend with SRV EF. [15] It is in agreement with our results, as we indicated a larger RV cavity and a higher RV mass among LGE positive patients. We also demonstrated the difference in SRV EF between LGE positive vs LGE negative patients. While small in absolute number (3% difference) it was very close to the value observed by Rydman et al, (2% difference), who demonstrated the prognostic value of SRV EF in an univariate Cox model. [31] While the difference seems to be clinically important, it should be noted that the cited values are very close to or even below intraobserver/interobserver reproducibility. [31, 32] Additionally, we showed an increased level of CITP when the LGE test was positive. This is in contrast with the data from Dos et al., where LGE positive patients did not have a significantly increased CITP level. [20] While there is no straightforward explanation for this difference it should be noted that the percentage of LGE positive CMR studies was substantially smaller in our study, as compared to Dos et al. (10% vs 27%). The incidence of LGE positive CMR studies among patients with SRV varies substantially. [12–14] Correlation of CITP levels with LGE might be dependent on the extent of fibrosis.

It is also noteworthy that NT-pro-BNP was significantly increased in patients with systemic right ventricle. In most previously published studies the levels of natriuretic peptides were higher in SRV patients compared with controls even when no signs or symptoms of heart failure were present. [33, 34] In accordance with former studies we confirmed a negative correlation between brain natriuretic peptide levels and SRV function. Contrary to NT-pro-BNP, aldosterone levels were comparable to healthy controls. It is well known that plasma concentration of aldosterone relates closely to the NYHA functional class and clinical indices of disease severity, while our cohort of patients was in good clinical condition (99% in NYHA class I or II). [35]

The added value of our work is that besides well proven predictors of outcome (SRV EF and NYHA class) we also found TIMP-1 and MMP9 as outcome predictors. Little is known about CTB’s prognostic value in cardiovascular diseases and no data is available from congenital heart disease patients. Most of the data point to the value of PIIINP and CITP in patients with heart failure, after myocardial infarction, dilated cardiomyopathy or hypertension. [36] Frantz et al. reported TIMP-1 as an independent predictor of all-cause mortality risk in patients with chronic heart failure. [37] Serum MMP-9 and TIMP-1 levels were associated with a risk of all-cause mortality in the prospective cohort study done by Hansson et al. TIMP-1 levels were mainly related to the risks of cardiovascular mortality and stroke. [38]

### Study limitations

Although the study group was relatively small it is the largest one concerning CTB among D-TGA patients. We analyzed biomarkers obtained from peripheral blood. For ethical reasons, it was not possible to sample myocardial biopsies or to investigate blood samples from the coronary sinus, which could be beneficial. Myocardial extracellular volume measurements with CMR T1 mapping techniques correlate with myocardial collagen volume fraction determined in myocardial samples of the left ventricle. Although the technique is evolving rapidly, assessment of right ventricular free wall with T1 mapping remains a challenge.

## Conclusions

In summary, while our cohort of patients with D-TGA was in good clinical condition, we demonstrated a pattern of altered collagen turnover and neurohormonal activation adversely correlated with indices of systemic right ventricular function and remodeling and assessed both by echocardiography and CMR. Even more importantly some of CTB were associated with an adverse clinical outcome in these patients.

These data provide the basis for the development of new diagnostic and therapeutic strategies in patients with SRV. However, because this is one of the first studies to investigate these relationships, further research is necessary.

## Supporting information

S1 Data Data underlying this study. (XLS)
